# Abscess after cardiac pacemaker implantation: A case report

**DOI:** 10.1002/ccr3.7639

**Published:** 2023-08-07

**Authors:** Joanna Rybak‐d'obyrn, Natalia Joanna Machoń, Julia Alicja Lewandowska, Agnieszka Owczarczyk‐Saczonek, Waldemar Placek

**Affiliations:** ^1^ Department of Dermatology, Sexually Transmitted Diseases and Clinical Immunology University of Warmia and Mazury Olsztyn Poland; ^2^ Medical Faculty University of Warmia and Mazury Olsztyn Poland

**Keywords:** abscess, cardiac pacemaker, CIED, *Staphylococcus aureus*

## Abstract

This is a report of one of the most serious complications of the cardiac pacemaker implantation ‐ infection of the implanted system. We present the case, which was misdiagnosed at the beginning and after cardiological consultation it was decided to immediately remove the peacemaker and transfer the patient to the Cardiological Department.

## Graphical abstract

Chest X‐ray of the patient, which shows pleural fluid in plaural cavities indicated the presence of inflammation.

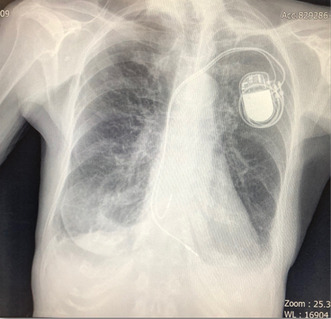

## 1 INTRODUCTION

Abnormalities in the activation or beating of the myocardium are called cardiac arrhythmias. It can be treated pharmacologically with and the implantation of a cardiac pacing system.^1^ These stimulating systems include pacemakers, implantable cardioverter defibrillators (ICDs), and cardiac resynchronization therapy (CRT) devices.^2^ The generator of peacemakers is placed in the left subclavian region in a lodge under the fascia of the pectoralis major muscle. The leads are inserted through the saphenous, axillary, or subclavian vein into the venous system, and the ends of the leads are pinched in the right heart cavities or branches of the coronary sinus.^3^ Pacemaker implantation is performed in patients meeting the American College of Cardiology and American Heart Association (ACC/AHA) guidelines.^4^ Despite the successes of electronic pacemakers, significant shortcomings related to the mechanical design of the device itself persist.^5^

In addition, pacemaker therapy, due to its invasive nature, is still associated with significant peri‐ and postprocedure complications. They are divided into immediate‐, intermediate‐, and long‐term ones.^6^ These complications include pocket (i.e., pacemaker bed) bleeding, pneumothorax, cardiac tamponade, electrode displacement, deep vein thrombosis, infections, and electrode or device failures.^7^ The pocket, systemic, associated endovascular leads, and the valves infection can be both intermediate and late time complications. At an earlier time, they are most often a result of an acute hematoma. Later the risk is higher for people with diabetes, heart failure, renal failure, corticosteroid use, postoperative hematoma, lack of antibiotic prophylaxis, oral anticoagulation, previous cardiac implantable electronic device (CIED) infection, and generator change.^6^ In this article, we report a case of skin infection and, more specifically, an abscess in the region of the pacemaker projection in a 90‐year‐old female patient.

## 2 CASE REPORT

A 90‐year‐old female patient was admitted to the Department of Dermatology, Sexually Transmitted Diseases and Clinical Immunology in Olsztyn in May 2022 because of erythema of the left upper chest. The first symptoms appeared 6 days before admission in the form of high fever, chills, significant weakness, followed by the appearance of painful, well‐demarcated erythema and swelling of the left upper chest.

**FIGURE 1:**
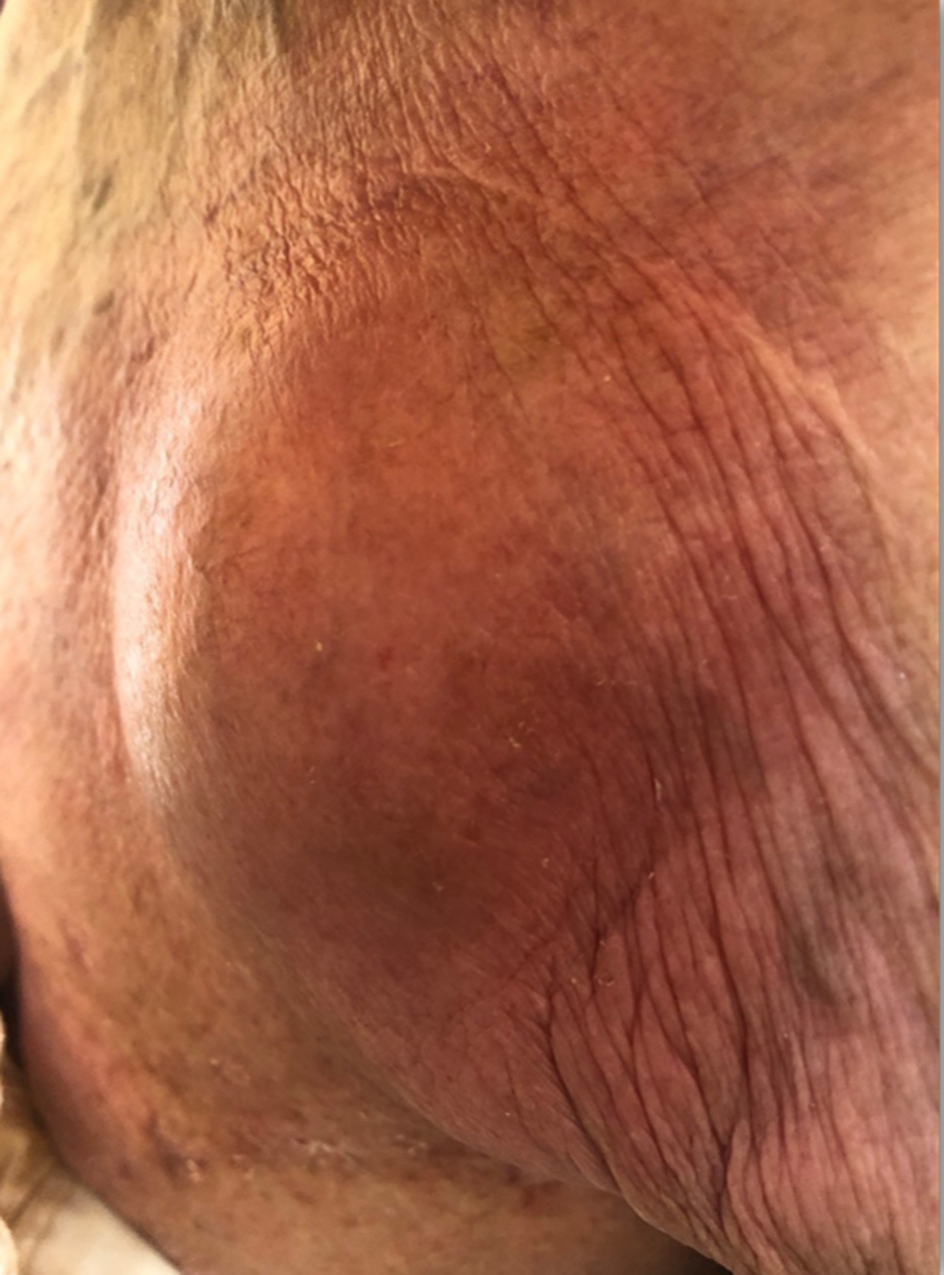
Severely painful during palpation, well‐demarcated, vivid‐red swelling, and erythema present in the left upper chest area. The infiltrate is located above and around the pacemaker, the skin above it is smooth, tense, and shiny.

The patient was initially treated on an outpatient basis with amoxicillin and paracetamol, and clindamycin was later added. However, due to the onset of nausea, vomiting, persistence of severe soreness of the left side of the chest and fever above 38°C, the patient was admitted to the Department of Dermatology. She had a history of postmastectomy and left‐sided radiation therapy for breast cancer (30 years ago), a DDD pacemaker implantation for bradycardia (2015), ischemic heart disease, colonic diverticulosis, and gastric ulcer disease. The patient had been taking medications such as molsidomine, nebivolol, rivaroxaban on a regular basis. The day before admission to the clinic, the patient was consulted by a cardiologist to check the pacemaker at the cardiology outpatient clinic ‐ the examination showed no abnormalities. On admission to the department, the general condition was average, asthenic physique, pale skin. The heart rate was steady at about 90/min, auscultatorically a loud systolic murmur was heard over the whole heart. Severely painful during palpation, well‐demarcated, vivid‐red swelling and erythema were present in the left upper chest area. The infiltrate was located above and around the pacemaker, the skin above it was smooth, tense, and shiny, with a fluctuant sensation when palpated (Figure 1).

**FIGURE 2:**
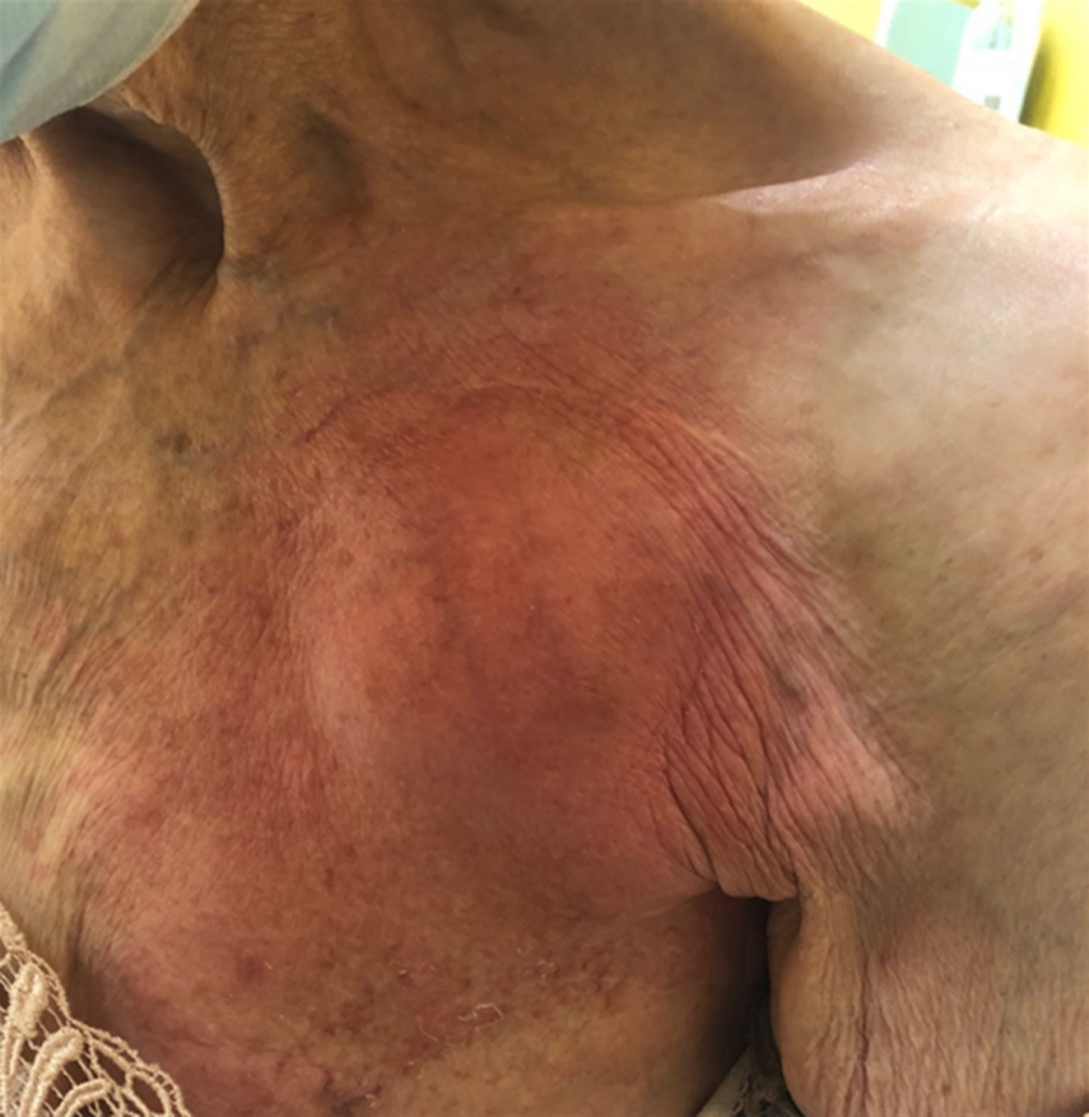
The skin of the left side of the chest was atrophic, with numerous discolorations (signs of late radiation reaction ‐ radiodermatitis).

In addition, a pitting oedema of the left upper limb was present, extending from the shoulder to the level of the midforearm. The skin of the left side of the chest was atrophic, with numerous discolorations (signs of late radiation reaction ‐ radiodermatitis) (Figure 2).

**FIGURE 3:**
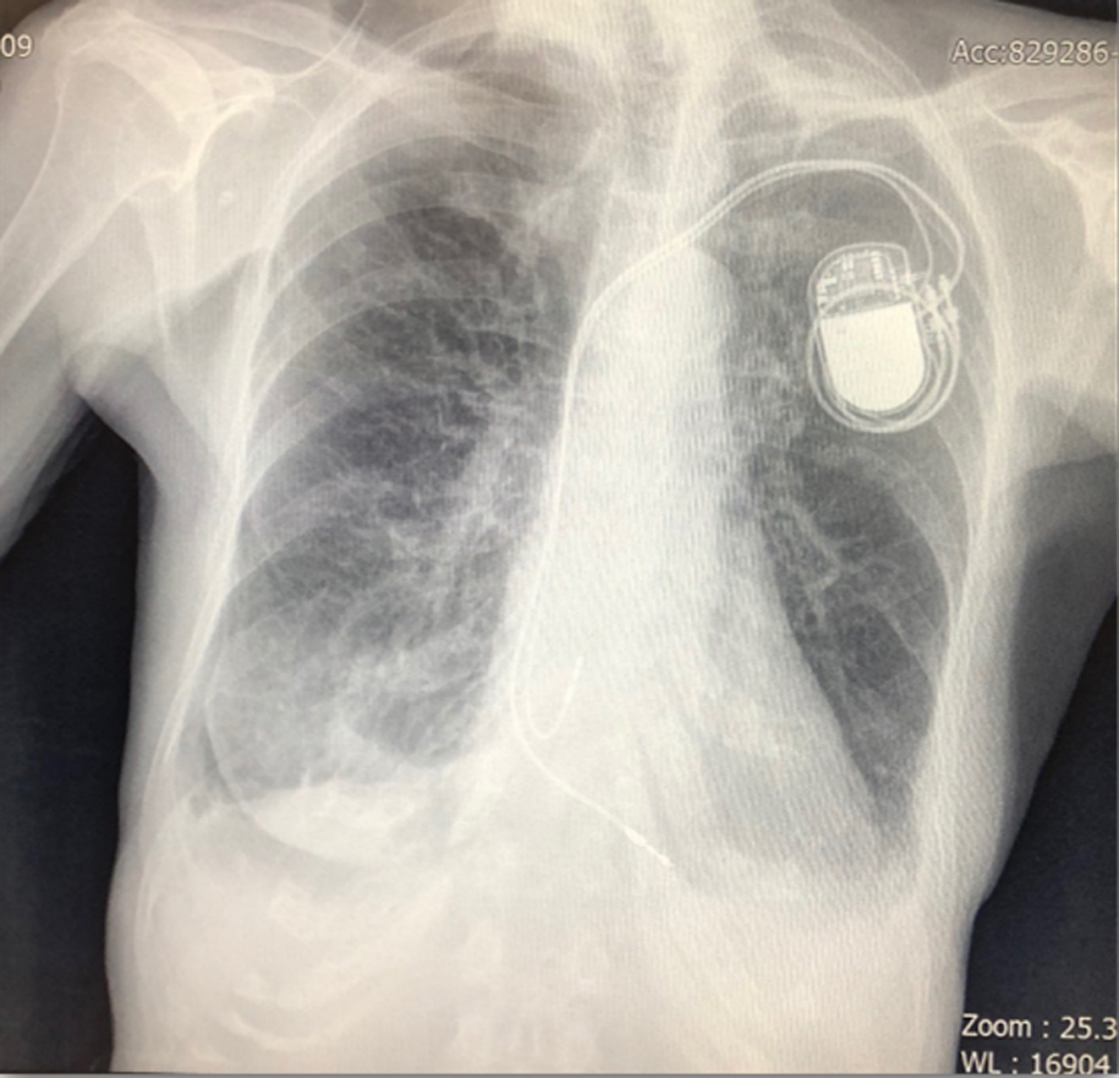
Chest X‐ray of the patient. It shows shadowed diaphragmatic and rib angles, which relates to pleural fluid in pleural cavities.

In laboratory tests on admission: White Blood Cells (WBC) = 10.33 10^3^/μL (norm <10.0 10^3^/μL), hemoglobin = 9.6 g/dL (norm: 12–15.5 g/dL), neutrophils = 8.59 10^3^/μL (norm: 0.8–1.77 10^3^/μL), high inflammatory parameters: C‐reactive protein (CRP) = 166.37 mg/L (norm <5), procalcitonin = 1.43 (norm <0.5). Blood cultures (aerobic and anaerobic) and urine cultures were negative. On chest X‐ray the diaphragmatic and rib angles were shadowed, which indicated pleural fluid in pleural cavities (Figure 3).

**FIGURE 4:**
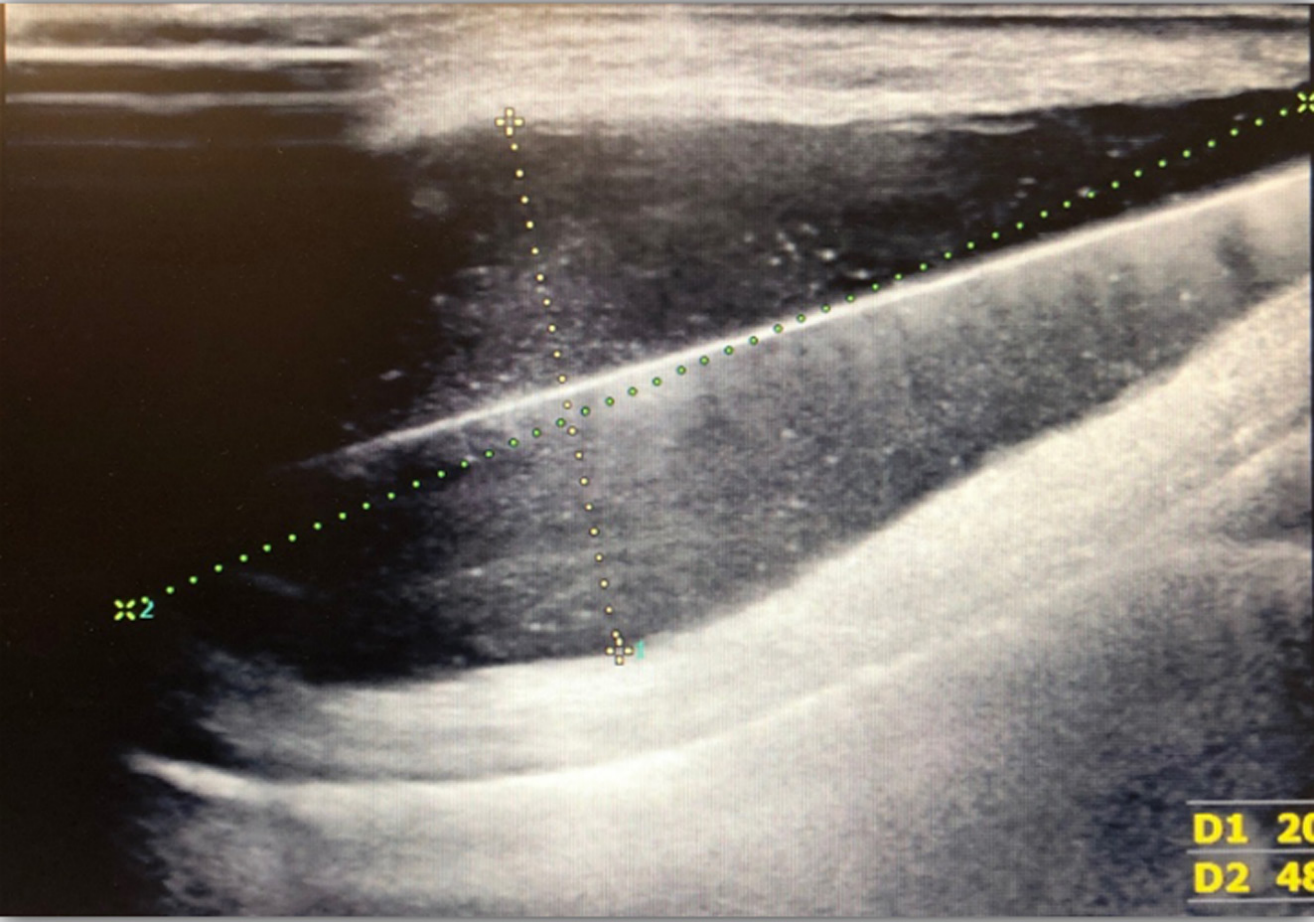
A superficial ultrasound of the tissues of the left subclavian region. It shows a 54 × 49 × 20 mm reservoir of echogenic fluid surrounding the internal pacemaker at a depth of 5 mm, the wall of the reservoir with visible vascularization.

A superficial ultrasound of the tissues of the left subclavian region showed a 54 × 49 × 20 mm reservoir of echogenic fluid surrounding the internal pacemaker at a depth of 5 mm. The wall of the reservoir had visible vascularization (Figure 4).

During hospitalization, a cardiological consultation was held, which confirmed that locally in the area of the pacemaker locus (in the left subclavian field) an inflammatory infiltrate with fluctuance was visible. Transthoracic echocardiography (TTE) showed no visible vegetations. However, tight aortic stenosis was visualized. An abscess of the pacemaker locus area was diagnosed. Treatment included antibiotic therapy (ceftriaxone and clindamycin), high positioning of the left upper limb (sling) and aluminum acetate gel packs. A gradual decrease in inflammatory parameters CRP = 115.61 mg/L, WBC = 9.86 10^3^/μL, procalcitonin ‐ normal and persistent anemia (stool for occult blood ‐ positive test) were observed. Due to a complication in the form of an abscess around the pacemaker locus, the patient was transferred to the Cardiology Department of the Olsztyn Regional Specialized Hospital for urgent removal of the cardiac pacing system. After the pacemaker was removed, a cardiac diagnostic was performed, after which reimplantation of the pacemaker was canceled due to the absence of further indications.

## 3 DISCUSSION

The implantation of a cardiac pacemaker is a method that has been used successfully to treat patients with cardiac arrhythmias for years. It has been improved over the years to be as safe, effective and cause as little complications as possible.^7^ In addition, pacemakers are increasingly becoming routine and, due to the aging population and thus increased cardiac morbidity, they are being inserted with increasing frequency. However, the number of complications is still significant. Their incidence varies according to the literature, and according to the type of device. The prospective multicenter cohort study FOLLOWPACE, published by Udo et al. showed that early complications (within 2 months) and, therefore, directly related to the surgical procedure, occurred in 12.4% of patients. However, late complications occurred in 9.2% of patients.^8^ In another study, published by Kiviniemi et al., the results were divided into a period of early complications up to 2 weeks and late ones from 2 weeks onward. Early complications occurred in 6.7% of patients, and late complications occurred in 7.2% of patients. Nevertheless, almost 70% complications occurred in patients within 3 months.^9^

The complication that developed in our patient was infection of the cardiac pacing system. The risk of CIED infection reaches 0.5%–1% within 6 to 12 months.^10^ Patients who develop this complication have symptoms, which are typical of inflammation ‐ swelling, redness and warming of the skin over the pacemaker projection. In addition, there may be systemic manifestations such as fever above 38 Celsius degrees, chills, night sweats, malaise, and weight loss. Septic shock usually occurs in less than 10% of patients.^11^

Konstantinos et al. summarized the most common risk factors for infection, which included diabetes, kidney disease, COPD, the use of corticosteroids, malignancies, heart failure and the use of anticoagulants.^12^ However, the PADIT study identified 5 nonmodifiable risk factors for CIED infection, which include prior procedures (P), young age (A), depressed renal function (D), immunocompromised (I), and procedure type (T).^13^ Tarakji et al. in the WRAP‐IT study divided risk factors into those related to the patient or device and those related to the procedure. Patient/device‐related factors included previous CIED procedures, history of atrial arrhythmia, geographic location, device type and lower body mass index. Procedure‐related factors included longer surgery duration, location of the implant, perioperative use of glycopeptide antibiotics, use of anticoagulants and/or antiplatelet drugs.^14^ According to these studies, in our patient, the possible causes of this infection were: the use of anticoagulants, ischemic heart disease, and low body mass index.

The etiological factors mentioned in the literature are various. In a study by Sohail et al., among 29 patients, the majority had infection caused by Staphylococcus aureus (38%) and coagulase‐negative staphylococci (31%). Other etiologic agents were Gram‐negative rods, Fungi, Peptostreptococcus magnus, Polymicrobial, and no microorganisms were cultured in 10%.^15^ However, in another article published by Shah et al. out of 47 cases, 38.3% of the cause was *Staphylococcus aureus*, followed by 10.6% *Pseudomonas*, 8.5% *E. coli* and 6.4% *Klebsiella*.^16^ Based on the literature, it is the Staphylococcal species that is the most common microbial cause of infection in patients with an implanted cardiac pacing system, which also confirms the case of our patient.^14^ Chamis et al. conducted a study among patients with S. aureus bacteremia and a pacemaker to determine the incidence of cardiac device infection in these patients. The overall incidence of cardiac device infection was about 45%. Tissue infection was the most common presumed source of *S. Aureus* bacteremia (SAB), occurring in more than half of the 33 patients studied. 30.3% of patients had an endovascular device source, while the primary source of bacteremia in 6 patients (18.2%) was a cardiac device. Patients with early SAB (that occurred up to 1 year after surgery) were significantly more likely to have a cardiovascular device as the source of bacteremia, while patients with late SAB (more than 1 year) were significantly more likely to have a tissue source of bacteremia.^17^

In determining the cause of infection, taking blood culture is necessary. In addition, it is worth performing a transthoracic echocardiography (TTE) to determine the involvement of the leads or heart valves and to determine the size of the vegetation. Laboratory diagnosis plays an important role to determine the presence of infection. Therefore, leukocyte count, C‐reactive protein (CRP), procalcitonin and erythrocyte sedimentation rate should be tested. However, it should be noted that laboratory markers of inflammation may be normal, even if the infection has been clearly identified.^11^

Management of this infection includes antibiotic therapy, transvenous lead extraction and surgical extraction, and potential reimplantation. Initial empirical antibiotic therapy in superficial infections, as well as isolated local pocket infections, should be initiated immediately after sample collection, include the typical bacterial spectrum, and take into account local resistance. Any infection involving the device is an indication for complete removal. Microbiological analysis of electrode tips and swabs obtained intraoperatively is necessary so that targeted antibiotic therapy can be initiated if a pathogen is detected. The indication for reimplantation needs to be verified, as 14%–33% of patients do not require implantation of a new device after electrode removal. If reimplantation is indicated, it is performed on the side opposite the previous explantation.^11^ In the reported case, reimplantation was discontinued because there was no need for further cardiac stimulation. This is also an interesting observation, as routine close cardiac controls and verification of the subsequent necessity of prolonged peacemaker use would have prevented any potential later complications. In addition, contributing to this is the fact that check‐ups of CIEDs are performed infrequently (once a year), and these days, even more often remotely, obtaining information only on the technical aspect of the devices' functioning, without examining the patient's skin. And although cardiac devices are used on a regular basis, still many doctors of various specialties forget about the possibility of them causing skin complications years later. Therefore, in patients with an implanted pacemaker, even during a routine dermatological examination, it is worth assessing the condition of the skin and subcutaneous tissue area above the device.

It is also worth mentioning that not every skin lesion in the area of the pacemaker's projection necessarily signifies an infection related to the pacing system. Korantzopoulos et al. summarized, based on a case series, other possible skin lesions in this area that, at first glance, could mimic an infection of the cardiac implantation electronic device. Such causes may include superficial cellulitis, herpes zoster over the pocket area, bruising over the pocket a long time after implantation in a patient taking oral anticoagulation and contact dermatitis due to prolonged postoperative application of povidone iodine.^18^

## 4 CONCLUSIONS

The frequency of complications associated with implantation of cardiac pacing systems is still significant. Although most of them occur in the early period after implantation, late complications and the potential risk of death should also be kept in mind. In this way they can be minimized. On the other hand, it is also worth remembering the potential other causes of the presence of inflammation in the pacemaker's projection and not rashly deciding to remove it, in order not to expose the patient to the risks associated with the operation itself as well as to unnecessary reimplantation.

## AUTHOR CONTRIBUTIONS

**Joanna Rybak‐d'Obyrn:** Conceptualization; data curation; formal analysis; investigation; methodology; resources; validation; visualization; writing – original draft. **Natalia Joanna Machoń:** Conceptualization; data curation; formal analysis; investigation; methodology; resources; validation; visualization; writing – original draft. **Julia Alicja Lewandowska:** Conceptualization; data curation; formal analysis; investigation; methodology; resources; validation; visualization; writing – original draft. **Agnieszka Owczarczyk‐Saczonek:** Conceptualization; supervision; writing – review and editing. **Waldemar Placek:** Conceptualization; supervision; writing – review and editing.

## FUNDING INFORMATION

None to declare.

## CONFLICT OF INTEREST STATEMENT

None to declare.

## ETHICS APPROVAL AND PATIENT CONSENT STATEMENT

Written consent for the publication of this case report was obtained from the patient. Approval for a case report by the institutional ethics committee is not required.

## Data Availability

The authors confirm that the data supporting the findings of this study are available within the article.
